# Complementary Therapies for Diabetic Foot Ulcer Healing Among Patients in Asia: Scoping Review

**DOI:** 10.2196/76301

**Published:** 2026-03-19

**Authors:** A Dian Miranda Yusran P, Saldy Yusuf, Herlina Burhan, Muhammad Jufri Taming

**Affiliations:** 1 Faculty of Nursing, Hasanuddin University, JL Perintis Kemerdekaan KM 10, Makassar, 90245, Indonesia, 62 81241841800; 2 Indonesian Diabetic Foot Care Research Group, Faculty of Nursing, Universitas Hasanuddin, Makassar, Indonesia

**Keywords:** diabetic foot ulcer, complementary therapies, wound healing, Asia, PRISMA, Preferred Reporting Items for Systematic reviews and Meta-Analyses

## Abstract

**Background:**

Diabetic foot ulcers (DFUs) are a severe complication of diabetes mellitus that can lead to amputation and mortality. Conventional treatments may be insufficient, leading to an interest in complementary therapies such as herbal medicine, acupuncture, maggot debridement therapy, and biological therapies. These approaches are widely used in Asia, yet their effectiveness and integration into clinical practice remain underexplored.

**Objective:**

The scoping review aimed to map the types of complementary therapies used for DFU healing in Asia and evaluate their reported effectiveness, implementation challenges, and opportunities for integration into conventional care.

**Methods:**

A scoping review was conducted using the PRISMA-ScR (Preferred Reporting Items for Systematic reviews and Meta-Analyses for Scoping Reviews) framework and the methodology of Arksey and O’Malley. Articles were sourced from the PubMed, Scopus, and ProQuest databases, covering studies published from 2014 to 2024. The population, concept, and context model guided the selection of studies, focusing on patients with DFUs, complementary therapies, and the Asian region.

**Results:**

Eight studies met the inclusion criteria. The most commonly used therapies included herbal treatments (eg, traditional Chinese medicinal foot soaks and *Teucrium polium*), biological therapies, including maggot debridement therapy and platelet-rich fibrin with hyaluronic acid), physical therapy (acupuncture), and psychological therapies (music therapy). Topical *T polium* significantly reduced wound size, and platelet-rich fibrin combined with hyaluronic acid increased vascular endothelial growth factor levels while reducing inflammation. Music therapy lowered the diabetes-related distress score. Despite these promising results, challenges remain, including a limited number of large-scale randomized controlled trials, regulatory barriers, and cultural perceptions affecting therapy acceptance.

**Conclusions:**

Complementary therapies are promising adjuncts for DFU management in Asia, where traditional medical practices are prevalent. Multidisciplinary collaboration between health care providers, policymakers, and traditional practitioners is essential for safe and effective integration. Further well-designed randomized controlled trials are required to confirm the efficacy of these therapies and inform evidence-based policies.

## Introduction

Diabetic foot ulcers (DFUs) are a severe complication of diabetes mellitus that can have significant health consequences, including amputation and increased mortality. Their highly complex pathophysiology involves a combination of ischemic neuropathy and infection, which hinders wound healing. Furthermore, prolonged hyperglycemia, hypertension, and hyperlipidemia contribute to oxidative stress and impaired blood flow, making DFUs particularly challenging to heal [1]. The lifetime incidence of foot ulceration among people with diabetes is estimated at 19% to 34%, and this burden is projected to increase due to longer survival and more complex comorbid conditions [2]. A systematic review estimated the global prevalence of DFUs to be approximately 6.3%, while a local study in Pakistan reported a prevalence of 16% among patients with diabetes [3]. Moreover, DFUs are associated with an increased risk of lower-extremity amputation and higher mortality, particularly among older adults and individuals with comorbid conditions [4]. Given the high prevalence and serious complications of DFUs, a comprehensive treatment approach is essential. In addition to conventional medical therapy, various adjunctive interventions have been developed to support wound healing, including complementary therapies, which have garnered increasing attention in the management of DFUs.

The integration of complementary therapy in the management of DFUs in Asia faces various challenges that may affect its effectiveness and acceptance, one of which is a lack of understanding and knowledge about complementary therapies among patients and health care professionals. In recent years, the incorporation of complementary therapy into DFU management has gained increasing attention, as these approaches can be used alongside conventional medical treatments and improve patient outcomes [5]. Traditional medicine plays a significant role in DFU management in Asia, with traditional Chinese medicine (TCM) and Ayurveda being widely practiced. These approaches have been shown to offer benefits in wound healing and infection control through herbal medicine, acupuncture, and dietary modifications to accelerate the healing process and restore balance to the body [6]. Recent studies have indicated that specific TCM formulations, such as *shengji* ointment, can significantly enhance the healing rate of DFUs, highlighting the potential for integrating these therapies into conventional treatment protocols [7]. Similarly, Ayurvedic practices have been reported to reduce infection rates and improve the overall quality of life of patients with DFUs [8]. Despite the promise of complementary therapies to support DFU healing, challenges remain in their integration into clinical practice. Therefore, further research and evidence-based approaches are necessary to ensure the safety, efficacy, and acceptance of these therapies for the holistic management of DFUs.

Complementary therapy is essential for health care management, including in the context of patients with DFUs in Asia. Research indicates that although many patients are interested in complementary therapy, they often lack adequate information regarding its effectiveness and safety [9]. Complementary therapies such as acupressure and massage effectively reduce pain and improve patient quality of life; however, many individuals remain uncertain about integrating these therapies with conventional medical treatments [10]. A study revealed that among 653 patients with DFUs, 21.7% used topical alternative treatments, while 31.2% combined conventional and alternative therapies [11]. This trend underscores the importance of health care providers being aware of various treatment modalities that patients may use to complement standard medical care. The efficacy of some complementary therapies has been documented in recent literature, such as ozone therapy, which has shown promising results in DFU healing, as evidenced by case reports highlighting its success in high-risk patients [12]. As the number of patients using complementary therapy in DFU management continues to increase, a more inclusive and evidence-based approach is necessary to integrate these therapies into clinical practice. Collaboration between health care professionals and complementary therapy practitioners is crucial to ensure that patients receive maximum benefits safely and effectively.

Complementary therapy in Asia for DFU healing has increased patient interest in alternative treatments rooted in local medical traditions. However, challenges regarding regulation, education, and integration with conventional medical care remain. Past findings highlight the importance of nutritional factors in DFU healing, which may encourage patients to seek nutrition-based complementary therapies aligned with their traditional medical practices [13]. In addition, the frequency of DFUs among patients with diabetes underscores the need for a multidisciplinary approach that incorporates complementary therapy despite existing integration barriers [14]. A study examining DFU prevalence in Ethiopia highlighted the importance of health education in improving patients’ understanding of foot care, which may incorporate complementary therapies rooted in local practices [15]. Although this study focuses on therapies delivered by nurses in person, it is also important to note that digitalized approaches to complementary therapies are now emerging. The use of apps based on TCM and mind-body practices has become a popular alternative, expanding the reach of these therapies to patients accessing health services via mobile devices [16]. However, to date, no studies have specifically examined the effectiveness of complementary therapy for DFU healing in Asia. The question remains as to how effective complementary therapies are for promoting healing of DFUs in this region. Therefore, the primary aim of this scoping review was to map the types of complementary therapies applied to DFU healing in Asia. The secondary aim was to evaluate their reported effectiveness and identify the challenges and opportunities for integration into clinical practice.

## Methods

We conducted a scoping review based on the PRISMA-Scr (Preferred Reporting Items for Systematic reviews and Meta-Analyses for Scoping Reviews) guidelines [17] to collect and summarize the literature on complementary therapies for DFU healing in Asia.

The methodology used in this review followed the 5-stage framework developed by Arksey and O’Malley [18]. This approach maps the available literature, identifies research gaps, and provides a comprehensive overview of the existing evidence. The methodological stages applied in this review include stage 1, formulating the research question; stage 2, identifying relevant studies; stage 3, selecting studies for inclusion in the review; stage 4, charting data from the selected studies; and stage 5, analyzing, synthesizing, and reporting the findings.

### Stage 1: Research Question

The research question in this review is as follows: How effective are complementary therapies in promoting the healing of DFUs in Asia?

### Stage 2: Relevant Studies and Search Strategy

This review focuses on complementary therapies for DFU. A comprehensive literature search was conducted across PubMed, Scopus, and ProQuest databases between May and September 2023. To ensure the inclusion of the most recent evidence, the search was updated on January 18, 2025. The search was restricted to full-text articles published in English between January 2014 and December 2024, in accordance with the predefined 10-year review period. The search strategy used Medical Subject Headings terms and relevant Boolean operators, adapted for each database. Detailed search strings for all databases are provided in Multimedia Appendix 1.

### Stage 3: Study Selection

Studies were screened based on predefined inclusion and exclusion criteria (Multimedia Appendix 2) in accordance with PRISMA-ScR guidelines [17]. All records were managed in Rayyan AI (Rayyan Systems, Inc) [19]. Duplicates were removed before title, abstract, and full-text screening.

### Stage 4: Data Extraction

Data were extracted to obtain key information from journal articles relevant to the research topic, including authors’ names and publication years, titles, research objectives, study methods, findings, and conclusions. This step aimed to facilitate the extraction of essential data required for the synthesis of the study.

### Stage 5: Thematic Summary and Key Findings

The literature findings were used to identify the results based on emerging keywords. This review analyzed all articles based on their titles, abstracts, and full texts, followed by a thorough examination. All analyzed articles contained information regarding complementary therapies for DFU healing in Asia.

### Ethical Considerations

This study was declared exempt by the institutional review board of Universitas Hasanuddin, as the analyzed articles only contained anonymized information or did not include any personally identifiable information.

## Results

### Effectiveness of Complementary Therapies

**Figure 1.**
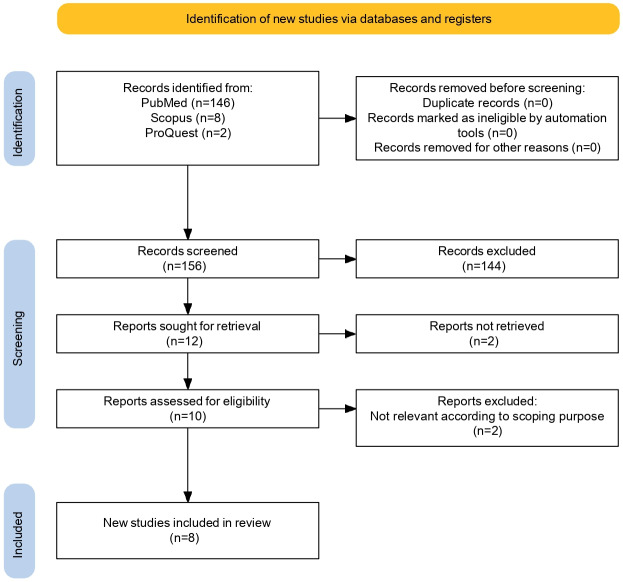
PRISMA-ScR (Preferred Reporting Items for Systematic reviews and Meta-Analyses for Scoping Reviews) flow diagram of the study selection process.

The review included a final total of 8 studies [6,20-26] (Figure 1). Several interventions demonstrated potential benefits for DFU management.

#### Herbal Therapies

A randomized controlled trial (RCT) on *Teucrium polium* ointment showed significantly greater wound size reduction and higher complete healing rates compared with placebo, with no notable adverse effects [22]. Another RCT protocol investigating a herbal foot bath decoction aims to evaluate its effects on inflammatory cytokines, including interleukin-6, as well as clinical outcomes in patients with diabetic peripheral neuropathy. [6].

#### Biological Therapies

Maggot debridement therapy (MDT) significantly increased microRNA-126 expression, enhancing angiogenesis and wound healing [21]. Similarly, the combination of advanced platelet-rich fibrin and hyaluronic acid resulted in higher vascular endothelial growth factor expression and lower interleukin-6 levels, indicating improved angiogenesis and reduced inflammation [26].

#### Psychological Therapies

A quasi-experimental study of music therapy found significant reductions in distress levels among patients with DFUs, highlighting the potential role of psychological interventions in improving patient well-being [25], which may ultimately facilitate the healing process for DFUs.

### Sociocultural Factors and Complementary and Alternative Medicine

Patterns of complementary and alternative medicine use were reported in both Indonesia and Thailand. In Indonesia, 54.3% of patients with type 2 diabetes used complementary and alternative medicine, with herbal medicine (100%), spiritual healing (68.3%), and massage (42.3%) being the most common, often recommended by family members. Key determinants included affordability, perceived safety, and cultural beliefs [20]. In Thailand, complementary and alternative medicine use was more prevalent among women and farmers, reflecting sociodemographic influences [23]. A qualitative study in rural China further revealed that traditional beliefs, limited access to health care services, and high treatment costs were significant barriers to timely medical care-seeking for DFUs [24]. These findings highlight the role of socioeconomic and cultural factors in shaping health behaviors and care choices among Asian populations with diabetes. In addition, DFUs impose a substantial economic burden in the region: in Iran, the economic impact was estimated at US $8.7 to $35 billion, representing 0.6% to 2.4% of the national GDP, whereas in Indonesia, in-hospital treatment costs for DFUs were Rp 65 million per episode (approximately US $3800), posing a considerable financial strain on patients and families [27,28].

### Study Characteristics, Implementation Challenges, and Emerging Models

**Figure 2.**
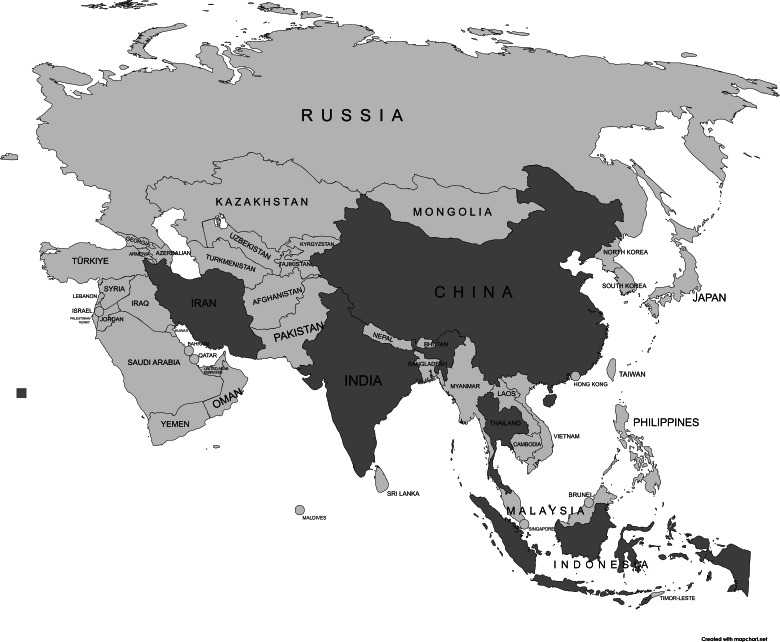
Geographic distribution of included studies on complementary and alternative therapies for diabetes and diabetic foot ulcers across Asia. Map generated using MapChart (Minas Giannekas) [29].

The included studies were conducted across several Asian countries: 2 in Indonesia [20,26], 3 in China [6,21,24], and 1 each in India [25], Thailand [23], and Iran [22], as illustrated in Figure 2. Settings varied, with most studies conducted in hospitals (n=3) [21,22,26], followed by primary health care centers (n=1) [23] and traditional hospitals (n=1) [6], as well as at a tertiary hospital (n=1) [25] and in rural communities (n=2) [20,24]. Study designs included 3 RCTs [6,22,26], 2 cross-sectional surveys [20,23], 1 quasi-experimental studies [25], 1 experimental (pre–post clinical study) [21] and 1 qualitative study [24]. Sample sizes ranged from 15 participants (in a qualitative study) to 628 participants (in a cross-sectional survey).

The key characteristics of the included studies are summarized in a synthesis grid (Table 1). Despite promising findings, the studies highlighted challenges in integrating complementary therapies into DFU management. These included small sample sizes, limited standardization of interventions, and variability across health care settings. At the same time, emerging models such as nurse-led telehealth and mobile health interventions showed potential for enhancing patient education and self-care practices. However, their clinical effectiveness in DFU outcomes requires further validation.

**Table 1.** Synthesis grid of included studies.

Authors (year)	Country	Study design	Sample size, n	Intervention/therapy	Main outcomes
Fan et al [6] (2018)	China	Randomized controlled trial	640	Foot bath decoction and placebo foot bath (traditional Chinese medicine)	Primary outcome: change in Toronto Clinical Scoring System score. Secondary outcomes: nerve conduction velocity, blood glucose (fasting blood glucose, postprandial blood glucose, hemoglobin a_1C_), blood lipids (total cholesterol, triglycerides, high-density lipoprotein, low-density lipoprotein), inflammatory cytokines (tumor necrosis factor-α, interleukin-6, C-reactive protein), quality of life (EQ-5D), and traditional Chinese medicine symptom scores.
Sari et al [20] (2021)	Indonesia	Cross-sectional	628	Assessment of self-reported CAM^a^ use (herbal medicine, spiritual practices, massage, honey	CAM use: 54.3% (herbal 100%, spiritual 68.3%, massage 42.3%); predictors: affordability (OR^b^ 4.59, 95% CI 3.01‐7.01), safety (OR 2.04), perceived effectiveness (OR 1.75).
Zhang et al [21] (2017)	China	Experimental (pre–post clinical study)	60	MDT^c^	Patients with diabetic foot ulcers had significantly lower microRNA-126 levels compared with patients without them (*P*=.001). Following MDT, microRNA-126 levels significantly increased (*P*=.04).
Fallah Huseini et al [22] (2024)	Iran	Randomized controlled trial	70	*Teucrium polium* ointment	Wound size decreased significantly from 3.52 (SD 1.47) cm^2^ to 0.717 (SD 0.19) cm^2^ in the intervention group, compared with a reduction from 3.21 (SD 1.67) cm² to 1.63 (SD 0.72) cm^2^ in the placebo group (*P*=.0001). Complete wound healing was achieved in 51.7% of patients in the intervention group versus 15.4% in the placebo group (*P*=.001)
Wanchai and Phrompayak [23] (2016)	Thailand	Cross-sectional	508	Self-reported use of CAM	CAM use was higher in women (*P*=.02) and farmers (*P*=.007).
Zhu et al [24] (2024)	China	Qualitative (interviews)	15	Exploration of sociocultural factors influencing wound care–seeking behavior	Barriers included traditional beliefs, limited access, and high treatment costs.
Ullas et al [25] (2023)	India	Quasi-experimental	30	Music therapy intervention	The distress score significantly decreased, from 3.7 (SD 0.6) at baseline to 2.4 (SD 0.2) after the intervention (*P*=.04).
Kartika et al [26] (2021)	Indonesia	Randomized controlled trial	30	Advanced platelet-rich fibrin combined with hyaluronic acid	Vascular endothelial growth factor levels increased significantly on day 3 (*P*=.003) and day 7 (*P*<.001), while interleukin-6 levels decreased significantly on day 7 (*P*=.02).

a CAM: complementary and alternative medicine.

b OR: odds ratio.

c MDT: maggot debridement therapy.

## Discussion

### Effectiveness of Complementary Therapies

Our scoping review highlights the potential role of complementary therapies in DFU management, including herbal interventions such as TCM foot bath decoction [6] and *Teucrium polium* [22], demonstrated anti-inflammatory and pro-angiogenic properties, consistent with their use in Asian traditional medicine practices [6,21,26]. Despite encouraging results, most studies were limited by small sample sizes, inadequate randomization, and a lack of standardized dosages, reducing the generalizability of findings. Biological therapies, including MDT and platelet-rich fibrin combined with hyaluronic acid, also showed promising effects in enhancing angiogenesis and reducing inflammation. However, evidence remains inconsistent across studies, with some RCTs questioning the superiority of MDT over standard care [21]. These findings underscore the need for rigorously designed clinical trials to establish safety, efficacy, and standardized protocols for complementary therapies in DFU care.

### Sociocultural and Economic Context in Asia

Sociocultural and economic contexts strongly influence treatment-seeking behaviors and the adoption of complementary and alternative medicine in Asia. In Indonesia and Thailand, complementary and alternative medicine use is widespread among patients with diabetes, primarily driven by affordability, cultural beliefs, and family influence [20,23,30]. In rural China, traditional beliefs, limited access to health care services, and high treatment costs were reported as significant barriers to timely DFU care [24]. These patterns reflect broader socioeconomic challenges in many Asian countries, where limited insurance coverage and high out-of-pocket costs make conventional DFU treatments less accessible. Consequently, patients often turn to complementary and alternative medicine as a perceived affordable and culturally acceptable alternative. Addressing these structural factors is essential to improving equity in DFU management and to ensuring that evidence-based complementary therapies are safely integrated into clinical practice.

### Holistic and Emerging Models of Care

Psychological and digital health interventions offer additional opportunities to complement medical and surgical approaches to DFU care. Music therapy, for example, was associated with significant reductions in diabetes-related distress, which may improve adherence to wound care and overall patient well-being [25]. Evidence from external studies also suggests that mindfulness and other behavioral interventions may enhance glycemic control and reduce distress, although their direct impact on DFU healing remains unclear [31]. Emerging models, including mobile health apps and nurse-led telehealth programs, have demonstrated feasibility in improving patient education, self-care practices, and remote consultation [30,32]. While clinical outcomes have not been consistently reported, these approaches highlight the potential for integrating psychological support and digital health innovations into holistic DFU management, particularly in resource-limited settings.

### Opportunities and Implications for DFU Management

Long-term clinical trials are necessary to assess the sustained effects of complementary therapies on wound healing and patient quality of life. Future studies should also explore the underlying biological mechanisms of herbal interventions to understand their therapeutic potential [22]. Investigating combined approaches, such as platelet-rich fibrin, hyaluronic acid, and antibiotics, may offer integrative treatment pathways for DFUs [26]. Collaboration between conventional clinicians and complementary therapy practitioners is vital to ensure evidence-based, safe, and practical implementation [20]. Additionally, enhancing patient education and public awareness about the realistic benefits and limitations of these therapies can promote more informed decision-making.

### Evaluation of Cost-Effectiveness

Economic evaluations of complementary therapies are required to compare their cost-effectiveness with that of conventional treatments. For instance, assessing the cost-benefit ratio of acupuncture for improving glycemic control or comparing MDT with antibiotic therapy in preventing amputations may help justify their integration into standard care [33,34]. Such analyses would provide valuable insights into the feasibility and scalability of incorporating complementary therapies into national health care systems.

### Recommendations

Future research is recommended to further investigate the effectiveness, biological mechanisms,and integration of complementary therapies in managing diabetes mellitus and DFUs. One critical direction for future studies involves conducting long-term clinical trials to evaluate the sustained effects of complementary therapies with a minimum follow-up period of 6 months or longer. This would provide a comprehensive understanding of their impact on wound healing, blood glucose control, and patient quality of life.

Qualitative studies of patients’ perceptions of complementary therapies are also important from social and cultural perspectives. Many patients with diabetes, particularly those in rural communities, tend to prefer traditional therapies to conventional medical treatments. Therefore, exploratory research should investigate the social, economic, and cultural factors that influence patients’ decisions to use complementary therapies. This can provide valuable insights into the factors driving therapeutic choices and contribute to the development of culturally sensitive health care interventions.

Lastly, cost-effectiveness studies of complementary therapies are necessary to compare their financial and therapeutic benefits with those of conventional treatments. Future research is expected to provide more robust scientific evidence regarding the efficacy, safety, and integration of complementary therapies for the management of diabetes and DFUs. This evidence-based approach will support the incorporation of complementary therapies into holistic and patient-centered care strategies, ultimately benefiting patients and the health care system.

### Critical Appraisal of Included Studies

Although we did not perform a critical appraisal because it is not mandatory in a scoping review, we observed that several included studies had limitations in study design. These included small sample sizes, a lack of randomization or blinding, and an absence of standardized outcome measures. The heterogeneity of study designs, ranging from case reports to quasi-experimental studies and RCTs, complicates the interpretation and generalization of our results. These limitations suggest that while the findings are promising, they should be interpreted cautiously and validated through further high-quality research.

### Opportunities and Implications

The integration of complementary therapies into clinical DFU care in Asia faces multiple barriers, as reflected by the studies included in this review. Regulatory frameworks for traditional and complementary therapies vary widely across countries, and are often incomplete or inconsistent, resulting in limited institutional support and ambiguous clinical guidelines. Furthermore, a lack of formal education and training for health care providers in evidence-based complementary practices contributes to uncertainty and resistance to implementation. Cultural perceptions also play a role, with some clinicians and patients viewing such therapies as informal or unscientific despite their profound historical and cultural relevance.

### Study Limitations

Although this scoping review indicates the potential of complementary therapies to support the healing of DFUs, there are several limitations in the current body of research. These limitations include a lack of long-term evidence and standardized therapeutic protocols, as well as challenges in integrating complementary therapies with conventional treatments. Therefore, further research using more rigorous clinical trial designs with larger sample sizes and comprehensive cost-effectiveness evaluations is warranted to ensure the safety and efficacy of complementary therapies in DFU management.

### Conclusion

Complementary therapies have significant potential for supporting the healing of DFUs in Asia; however, further research is necessary to confirm their efficacy and safety. Integrating complementary therapies into DFU management requires an evidence-based approach, more transparent regulatory frameworks, and comprehensive education for both health care providers and patients. With a thorough and evidence-based approach, complementary therapies can become part of a more holistic DFU treatment strategy, contributing to improved clinical outcomes and reducing the economic burden associated with diabetes complications.

## Supplementary material

10.2196/76301 Multimedia Appendix 1 Summary of literature search strategy—databases, keywords, and selection results.

10.2196/76301 Multimedia Appendix 2 Problem, concept, and context framework.

10.2196/76301 Checklist 1 PRISMA-ScR Checklist.

## Abbreviations

DFU: diabetic foot ulcer
MDT: maggot debridement therapy
PRISMA-ScR: Preferred Reporting Items for Systematic reviews and Meta-Analyses for Scoping Reviews
RCT: randomized controlled trial
