# Supplementary material for: Complementary Therapies for Diabetic Foot Ulcer Healing Among Patients in Asia: Scoping Review
Source: Asian Pac Isl Nurs J. 2026 Mar 19;10:e76301. doi: 10.2196/76301 (PMC13002009; doi:10.2196/76301)
Supplement: Multimedia Appendix 1 [file apinj-v10-e76301-s001.docx]

Table S1. Summary of literature search strategy, databases, keywords, and selection results.

| **NO** | **Data Base** | **Keywords** | **Initial Articles** | **Final Articles** | **Date Accessed** |
| --- | --- | --- | --- | --- | --- |
| 1 | PubMed | (((diabetic foot ulcer[Title/Abstract]) AND (Complementary therapy[MeSH Terms])) OR (("complementary therapy"[Title/Abstract] OR "alternative therapy"[Title/Abstract] OR "complementary and alternative medicine"[Title/Abstract] OR "holistic therapy"[Title/Abstract] OR "integrative medicine"[Title/Abstract] OR "traditional medicine"[Title/Abstract]))) AND ((China[Title/Abstract] OR Japan[Title/Abstract] OR "South Korea"[Title/Abstract] OR "North Korea"[Title/Abstract] OR Mongolia[Title/Abstract] OR Taiwan[Title/Abstract] OR Indonesia[Title/Abstract] OR Malaysia[Title/Abstract] OR Thailand[Title/Abstract] OR Philippines[Title/Abstract] OR Vietnam[Title/Abstract] OR Singapore[Title/Abstract] OR Myanmar[Title/Abstract] OR Cambodia[Title/Abstract] OR Laos[Title/Abstract] OR Brunei[Title/Abstract] OR "Timor-Leste"[Title/Abstract] OR India[Title/Abstract] OR Pakistan[Title/Abstract] OR Bangladesh[Title/Abstract] OR Sri Lanka[Title/Abstract] OR Nepal[Title/Abstract] OR Bhutan[Title/Abstract] OR Maldives[Title/Abstract] OR Kazakhstan[Title/Abstract] OR Kyrgyzstan[Title/Abstract] OR Tajikistan[Title/Abstract] OR Turkmenistan[Title/Abstract] OR Uzbekistan[Title/Abstract] OR "Saudi Arabia"[Title/Abstract] OR UAE[Title/Abstract] OR Israel[Title/Abstract] OR Iran[Title/Abstract] OR Iraq[Title/Abstract] OR Turkey[Title/Abstract] OR Jordan[Title/Abstract] OR Lebanon[Title/Abstract] OR Syria[Title/Abstract] OR Qatar[Title/Abstract] OR Oman[Title/Abstract] OR Bahrain[Title/Abstract] OR Kuwait[Title/Abstract] OR Yemen[Title/Abstract] OR Palestine[Title/Abstract])) | 4.767 | 146 | January 18, 2025 |
| 2 | ProQuest | abstract(diabetic foot ulcer or ulcer ) AND abstract(complementary or alternative therapies) AND abstract(china OR japan OR South Korea OR North Korea OR mongolia OR taiwan OR indonesia OR malaysia OR thailand OR philippines OR vietnam OR singapore OR myanmar OR cambodia OR laos OR brunei OR Timor Leste OR india OR pakistan OR bangladesh OR Sri Lanka OR nepal OR bhutan OR maldives OR Kazakhstan OR kyrgyzstan OR tajikistan OR Turkmenistan OR uzbekistan OR Saudi Arabia OR uae OR Israel OR iran OR iraq OR turkey OR Jordan OR lebanon OR Syria OR qatar OR oman OR Bahrain OR Kuwait OR Yemen OR palestine) | 24 | 2 | January 18 2025 |
| 3 | Scopus | diabetic AND foot AND ulcer AND complementary AND therapies AND ( china OR japan OR "South Korea" OR "North Korea" OR mongolia OR taiwan OR indonesia OR malaysia OR thailand OR philippines OR vietnam OR singapore OR myanmar OR cambodia OR laos OR brunei OR "Timor-Leste" OR india OR pakistan OR bangladesh OR "Sri Lanka" OR nepal OR bhutan OR maldives OR Kazakhstan OR kyrgyzstan OR tajikistan OR turkmenistan OR uzbekistan OR "Saudi Arabia" OR uae OR israel OR iran OR iraq OR turkey OR jordan OR lebanon OR syria OR qatar OR oman OR bahrain OR kuwait OR yemen OR palestine ) | 10 | 8 | January 18, 2025 |
