# Supplementary material for: Complementary Therapies for Diabetic Foot Ulcer Healing Among Patients in Asia: Scoping Review
Source: Asian Pac Isl Nurs J. 2026 Mar 19;10:e76301. doi: 10.2196/76301 (PMC13002009; doi:10.2196/76301)
Supplement: Multimedia Appendix 2 [file apinj-v10-e76301-s002.docx]

Article Eligibility Criteria

| Kriteria | Description |
| --- | --- |
| Problem | Diabetic foot ulcer |
| Concept | Complementary therapy |
| Context | Asia |
